# Limited Bacterial Diversity within a Treatment Plant Receiving Antibiotic-Containing Waste from Bulk Drug Production

**DOI:** 10.1371/journal.pone.0165914

**Published:** 2016-11-03

**Authors:** Nachiket P. Marathe, Sudarshan A. Shetty, Yogesh S. Shouche, D. G. Joakim Larsson

**Affiliations:** 1 Department of Infectious Diseases, Institute of Biomedicine, The Sahlgrenska Academy at the University of Gothenburg, Göteborg, Sweden; 2 Centre for Antibiotic Resistance Research (CARe) at University of Gothenburg, Göteborg, Sweden; 3 Laboratory of Microbiology, Wageningen University, Wageningen, The Netherlands; 4 Microbial Culture Collection (MCC), National Center for Cell Science, Pune, Maharashtra, India; MJP Rohilkhand University, INDIA

## Abstract

Biological treatment of waste water from bulk drug production, contaminated with high levels of fluoroquinolone antibiotics, can lead to massive enrichment of antibiotic resistant bacteria, resistance genes and associated mobile elements, as previously shown. Such strong selection may be boosted by the use of activated sludge (AS) technology, where microbes that are able to thrive on the chemicals within the wastewater are reintroduced at an earlier stage of the process to further enhance degradation of incoming chemicals. The microbial community structure within such a treatment plant is, however, largely unclear. In this study, Illumina-based 16S rRNA amplicon sequencing was applied to investigate the bacterial communities of different stages from an Indian treatment plant operated by Patancheru Environment Technology Limited (PETL) in Hyderabad, India. The plant receives waste water with high levels of fluoroquinolones and applies AS technology. A total of 1,019,400 sequences from samples of different stages of the treatment process were analyzed. In total 202, 303, 732, 652, 947 and 864 operational taxonomic units (OTUs) were obtained at 3% distance cutoff in the equilibrator, aeration tanks 1 and 2, settling tank, secondary sludge and old sludge samples from PETL, respectively. *Proteobacteria* was the most dominant phyla in all samples with *Gammaproteobacteria* and *Betaproteobacteria* being the dominant classes. *Alcaligenaceae* and *Pseudomonadaceae*, bacterial families from PETL previously reported to be highly multidrug resistant, were the dominant families in aeration tank samples. Despite regular addition of human sewage (approximately 20%) to uphold microbial activity, the bacterial diversity within aeration tanks from PETL was considerably lower than corresponding samples from seven, regular municipal waste water treatment plants. The strong selection pressure from antibiotics present may be one important factor in structuring the microbial community in PETL, which may affect not only resistance promotion but also general efficiency of the waste treatment process.

## Introduction

Waste water from antibiotic production often contains high levels of antibiotics, indicating risks for the promotion of antibiotic resistance inside the treatment plants or in contaminated environments [1]. Studies on penicillin and oxytetracycline production in China indicated an enrichment of antibiotic resistant bacteria and mobile genetic elements during biological treatment of antibiotic-contaminated waste [2, 3]. We have previously studied an industrial waste water treatment plant (WWTP) in Patancheru, India operated by Patancheru Environment Technology Limited (PETL) [4, 5, 6, 7, 8]. Treated effluent from PETL, which receives waste from a large number of production units, was contaminated by a range of drugs including broad spectrum fluoroquinolone antibiotics like ciprofloxacin, enrofloxacin and norfloxacin at very high concentrations [4]. Kristiansson et al (2011) demonstrated very high levels of resistance genes to several classes of antibiotics in sediment of the river where PETL used to discharge their effluent. Today, the effluent from PETL has been rerouted to another river system via an 18 km long pipeline [9], principally just moving the antibiotic pollution problem [10]. A culture-based study on bacteria thriving inside PETL revealed many highly multidrug resistant bacterial strains, particularly of the genera *Pseudomonas* and *Ochrobactrum* [8].

Although much research, rightly, has focussed on risk for promotion of antibiotic resistance, there is limited knowledge about the microbial community structure within WWTPs treating waste from antibiotic production [11]. Activated sludge (AS) treatment is the most commonly used technique in WWTPs for treating both municipal and industrial wastewater due to its high efficiency and low operational cost. During AS treatment of waste, complex populations of highly specialized bacteria play an important role in the removal of different organic pollutants and nutrients [12, 13, 14]. The presence of bacterial strains that have the ability to degrade incoming organic matter is critical for ensuring efficiency. During AS treatment, this is normally achieved by recycling part of the sludge accumulated at a later stage of the process and adding it back to spike the initial aeration tanks [12, 13, 14]. In the case of antibiotic containing waste, this in turn also would lead to selection of antibiotic resistant bacteria. Along with the selection of resistant strains, a general impact on the composition of the microbial communities of the AS treatment process would be expected due to inherent differences in antibiotic susceptibility between species. Such changes could, in turn, negatively affect the treatment efficiency for organic molecules other than the antibiotics themselves. Thus, it would be informative to explore the bacterial community composition in full-scale WWTPs treating antibiotic production waste.

Culture-dependent techniques have been widely used for studying bacteria present in WWTPs [12, 15]. As such approaches are limited to a minor, culturable part of the bacterial commuinities [16, 17], culture-independent approaches like denaturation gradient gel electrophoresis (DGGE), clone library preparation using 16S rRNA gene and fluorescence *in situ* hybridization (FISH) have been succesfully utilized for studying total bacteria flora in microbial ecosystems [11, 12]. More recently, 16S rRNA gene amplicon sequencing using high throughput sequencing technologies has been successfully applied to explore the bacterial diversity in a given sample [18, 19, 20]. These high throughput techniques allow the detection of low-abundant microorganisms and capture higher bacterial diversity compared to traditional 16S rRNA gene clone library or FISH analysis [20, 21].

The objective of the present study was to describe the bacterial taxonomic composition during different stages of treatment in a full-scale Indian WWTP (PETL) treating antibiotic-contaminated waste. For this purpose we applied Illumina Miseq based sequencing of 16S rRNA gene variable region V3. Further, we compared the bacterial diversity in PETL with similar, published data from 7 other WWTPs treating mainly municipal sewage [19].

## Materials and Methods

### Sample collection and DNA extraction

The samples were collected from PETL (N17° 32.396 E78° 14.590) at the same time as for a previous culture-based study of ours [8] where we also described sampling and DNA extraction procedures in more detail. Briefly, grab samples of water from the equilibrator (EQR), aeration tank no.1 (AER1), aeration tank no. 2 (AER2), settling tank (STL) and sludge samples from the secondary sludge (SS) and old dried sludge (OS) were collected in sterile containers. These samples were stored on ice and transported overnight to the National Center for Cell Science, in Pune, Maharashtra, India. The sampling was authorized by the Pollution Control Board of Andhra Pradesh and supervised by the manager of PETL.

The total genomic DNA was extracted from concentrated water samples and sludge samples using PowerSoil^TM^ DNA Isolation Kit (MoBio Laboratories Inc., Carlsbad, USA) according to manufacturer´s instructions. The extracted DNA was quantified using a Nanodrop spectrophotometer (J H Bio innovations, Secunderabad, India) and stored at -20°C until further use.

### Amplicon sequencing and filtering of raw reads

The V3 region of 16S rRNA gene was amplified from the extracted DNA using 341F and 518R primers as described previously [22, 23]. The V3 region of 16S rRNA gene was selected for the study as it has been previously used for Illumina-based microbial profiling [22]. The V3 region has a high taxonomic resolution and highest overall coverage compared to other variable regions of 16S rRNA gene [24, 25]. The samples were sequenced at SciGenom labs Hyderabad, India using Illumina Miseq platform, with 2×150 paired-end chemistry. The obtained reads were assembled using PANDASeq and reads with ambiguous bases after assembly were discarded [26]. The FASTQ files of assembled reads for each library were further filtered in Mothur v3.2 to retain reads between 150 bases and 160 bases with an average read length of 158.4 bases [27].

### OTU picking and Taxonomic assignment

We used phylotype approach for analysis and comparison of microbial diversity of different WWTP [28]. The operational taxonomic unit (OTU) picking was done using a closed-reference OTU picking protocol using the QIIME command: pick_closed_reference_otus.py with default parameters and using Greengenes database [29, 30]. Reads were assigned to OTUs based on their best hit to this database at greater than or equal to 97% sequence identity. Taxonomic assignment was done using RDP classifier based on the latest Greengenes taxonomy version 13.5 (http://greengenes.lbl.gov). The sequences from previous studies were obtained from NCBI Short Reads Archive database (accession number: SRA026842) and processed similar to the sequences generated in present study.

### Alpha Rarefaction and Beta Diversity Analysis

The alpha and beta diversity matrices phylogenetic diversity (PD whole tree), Good’s coverage, Chao 1 and observed species were calculated at an even sequencing depth (169,900 sequences per sample for comparisons of compartments within PETL and 16,770 sequences per sample for comparisons with other WWTPs). The weighted and unweighted Unifrac distances were calculated between all samples and results were visualized using the principal coordinate analysis in QIIME. The unweighted Unifrac distances were compared using two-sided Student's two-sample t-test and the nonparametric p-values were calculated using Monte Carlo permutations [31].

## Results and Discussion

### Bacterial community structure during different stages of AS treatment in PETL

**Fig 1:**
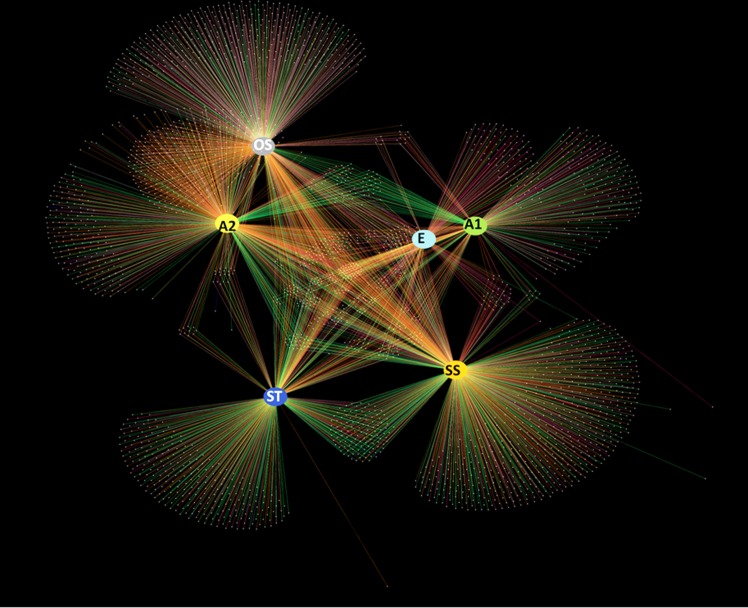
Network based representation of OTU clustering in different samples from PETL. The nodes represent each sample and the edges corresponding to specific phylum. The OTUs belonging to different phyla are colored with different colors. Abbreviations: E = equilibrator; A1 = aeration tank No. 1; A2 = aeration tank No. 2; ST = settling tank; SS = secondary sludge; OS = old dried sludge.

A total of 1,019,400 filtered sequences were analyzed and the library size of each sample was normalized to 169,900 sequences. The sequences have been deposited at MG-RAST server (http://metagenomics.anl.gov) under project PETL_16S_amplicon (project Id-18316). At 3% cut off, the EQR (equilibrator) had the lowest bacterial diversity indicated by the number of OTUs, Chao 1 index and the Shannon diversity index (Table 1). All the samples had a Good’s coverage of more than 0.99, suggesting that most of the bacterial diversity has been captured in the sequencing effort. The rarefaction curves represented in S1 Fig, demonstrate that bacterial OTU richness was lowest in the EQR, and then gradually increased along the AS treatment process. The differences in the bacterial community in samples from different stages are represented by the OTU network presented in Fig 1. A set of OTUs (5% to 60%) appeared to be unique to each sample.

**Table 1:** Diversity indices at different sampling points in PETL, generated by 16S amplicon sequencing (based on 169,900 sequences per sample).

Sample	Observed OTUs	Simpson's diversity	Chao 1 richness	Shannon diversity
EQR	202	0.05	377.3	0.29
AER1	303	0.40	500.7	1.63
AER2	732	0.73	1046.3	3.13
STL	652	0.69	1130.7	3.24
SS	947	0.92	1693.8	4.92
OS	864	0.67	1335.4	2.84

Legend: EQR = equilibrator; AER1 = aeration tank No. 1; AER2 = aeration tank No. 2

STL = settling tank; SS = secondary sludge; OS = old dried sludge

To the best of our knowledge, this is the most comprehensive study exploring the bacterial community of a full-scale WWTP treating wastewater from multiple drug production units. Deng et al [11] explored the microbial diversity of different steps of a Chinese WWTP treating wastewater from streptomycin production by Sanger sequencing of 16S rRNA clone libraries. Although the number of sequenced bacterial clones (n = 355) was limited, clear shifts in taxonomic composition was observed along the treatment process. At the streptomycin production waste treatment plant, recirculation of sludge was only applied to the last treatment step (oxidative ditch) [11]. Several studies on WWTP AS microbial community using high throughput sequencing technologies have focused only on a single step of the AS treatment, e.g. aeration tanks [19, 32] and occasionally sludge, influent and effluent samples [33] whereas comprehensive studies using high throughput sequencing on all the stages of AS treatment has previously been lacking.

**Fig 2:**
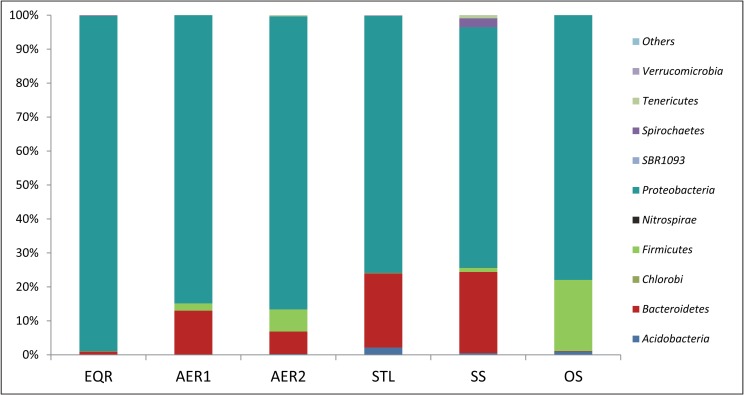
Phylum level bacterial diversity in PETL samples. Abbreviations: EQR = equilibrator; AER1 = aeration tank No. 1; AER2 = aeration tank No. 2; STL = settling tank; SS = secondary sludge; DS = dewatered sludge; OS = old dried sludge.

The phylum level bacterial diversity in the PETL samples is represented in Fig 2. *Proteobacteria* was the most dominant phyla in all the samples, ranging from 98.7% in EQR to 70.7% in SS (secondary sludge). This is consistent with previous studies reporting *Proteobacteria* to be the most dominant phyla in AS, followed by *Bacteroidetes* and *Firmicutes* [12, 13, 14, 19, 33, 33]. The phyla *Firmicutes* and *Bacteroidetes* were detected at very low levels in EQR but were present in significantly higher number in samples collected from aeration tanks, Fig 2. *Firmicutes* and *Bacteroidetes* are dominant phyla in human gut [34]. Higher abundance of these phyla in aeration tanks compared to EQR is consistent with the fact that human feces is added to the aeration tank in PETL, with the intention to maintain the biological activity of the AS treatment process [4, 5].

**Fig 3:**
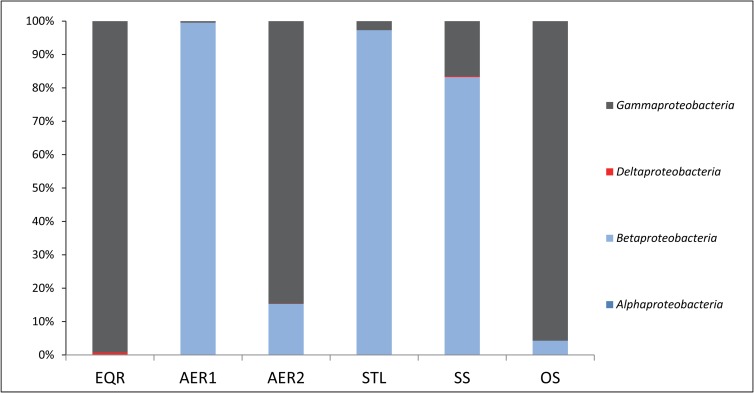
Class level distribution within the phylum *Proteobacteria* for PETL samples. Abbreviations: EQR = equilibrator; AER1 = aeration tank No. 1; AER2 = aeration tank No. 2; STL = settling tank; SS = secondary sludge; DS = dewatered sludge; OS = old dried sludge.

Within *Proteobacteria*, *Gammaproteobacteria* dominated EQR, AER2 and OS samples, while *Betaproteobacteria* dominated AER1, STL and SS samples (Fig 3). This suggests a possible influence of recycling secondary sludge on AER1 bacterial community, while AER2 bacterial community seems to be influenced mainly by the incoming waste from EQR. *Betaproteobacteria* and *Gammaproteobacteria* are known to play an important role in denitrification, removal of phosphorous and xenobiotic degradation from the waste and thus, represent important members of the AS microbial community [12]. *Deltaproteobacteria* were detected in low numbers in all the samples, *Alphaproteobacteria* were detected in only one sample and *Epsilonproteobacteria* were not detected in any of the samples. This is in some contrast to the study by Deng et al [11] where *Deltaproteobacteria* dominated during the earlier steps of the treatment process of streptomycin production waste.

At family level, *Alcaligenaceae*, *Comamonadaceae*, *Enterococcaceae*, *Flavobacteriaceae*, *Methylophilaceae*, *Porphyromonadaceae*, *Pseudomonadaceae*, *Streptococcaceae* and *Xanthomonadaceae* were the dominant bacterial families, representing at least 5% of the sequences in at least one of the samples Table 2. *Alcaligenaceae* and *Pseudomonadaceae* were the most abundant bacterial families in aeration tank samples. Previous studies on AS have shown bacterial families other than *Alcaligenaceae* and *Pseudomonadaceae*, to be dominant in aeration tanks of AS treatment [19, 32]. Although *Alcaligenaceae* and *Pseudomonadaceae* contain the genera *Alcaligenes* and *Pseudomonas* which are known to degrade a range of xenobiotics [35, 36, 37, 38], their dominance in aeration tanks is not in accordance with some earlier studies [12, 13, 14, 19, 32, 33]. In our previous culture-dependent study, we showed the presence of highly drug-resistant bacterial strains belonging to genera *Pseudomonas*, *Alcaligenes*, *Advenella*, *Bordetella*, and *Castellaniella*. All these strains were resistant to 15 to 31 antibiotics including fluoroquinolones [8]. It is quite likely that the high abundance of families *Alcaligenaceae* and *Pseudomonadaceae* in the PETL AS samples is partly due to the inherently resistant nature of bacteria from these families.

**Table 2:** The ten most abundant bacterial families found in samples from PETL, expressed as percentage of total sequences within each sample.

* *	EQR	AER1	AER2	STL	SS	OS
*Alcaligenaceae*	0.05	83.04	11.37	15.42	15.16	1.93
*Comamonadaceae*	0.00	0.00	1.29	0.71	15.66	1.36
*Enterococcaceae*	0.03	0.11	6.12	0.02	0.13	3.77
*Flavobacteriaceae*	0.00	0.00	0.28	2.76	8.44	0.00
*Methylophilaceae*	0.00	0.00	0.12	0.28	18.00	0.01
*Porphyromonadaceae*	0.57	12.57	0.05	0.08	2.13	0.00
*Pseudomonadaceae*	97.84	0.03	14.44	0.22	2.35	12.27
*Rhodocyclaceae*	0.00	0.00	0.00	54.12	0.62	0.00
*Streptococcaceae*	0.00	0.00	0.01	0.01	0.14	16.14
*Xanthomonadaceae*	0.00	0.26	0.00	0.69	5.41	0.24

Abbreviations: EQR = equilibratior; AER1 = aeration tank No. 1; AER2 = aeration tank No. 2; STL = settling tank; SS = secondary sludge; OS = old dried sludge

### Lower bacterial diversity in PETL

In order to understand the extent of bacterial diversity in PETL in context with regular sewage treatment plants, we compared the number of OTUs obtained in our study to previous reports on AS microbial diversity using NGS. We analyzed previously published amplicon sequencing data on aeration tanks of 7 different AS treatment plants in China using our analysis pipeline and compared it with aeration tanks from PETL [18, 19]. The details about the sampling sites are represented in S1 Table and Zhang et al. 2012 [19]. It should be stressed that these treatment plants mainly treat municipal waste and hence one would expect inherent differences in microbial composition within these plants compared to PETL. Moreover, the sequencing of these samples was done using V4, rather than the V3 region of 16S rRNA gene. Mizrahi-Man et al [39] investigated the efficiency of different variable regions of 16S rRNA gene for bacterial community profiling by NGS-based sequencing. They recommend the use of variable regions V3 or V4 for exploring bacterial communities in a given sample [39]. Similar observations were made by Vasileiadis et al showing that both V3 and V4 regions of 16S rRNA perform equally well for amplicon based bacterial community profiling [40]. Although, these studies have shown that there is no significant difference in microbial diversity while using V3 region or V4 region of 16S rRNA gene, some differences in microbial composition are unavoidable. Hence, in order to reduce the risk of making conclusions that are mainly due to the different amplicon strategy used, we limited our comparison below just to the number of OTUs detected and did not compare the bacterial composition per se.

**Fig 4:**
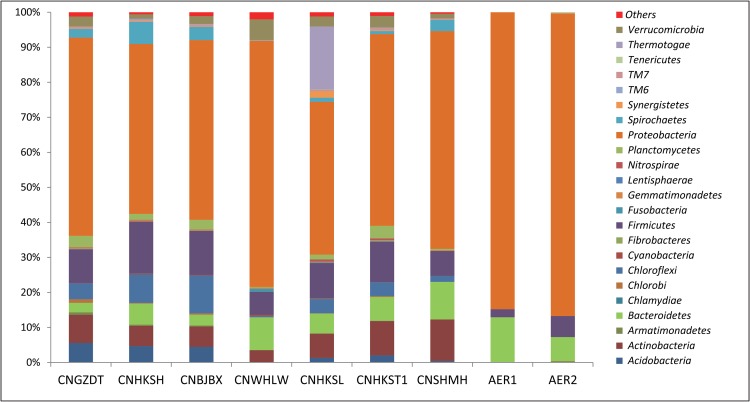
Microbial diversity of aeration tanks from seven regular municipal sewage treatment plants (not known to treat pharmaceutical industry waste) and the two aeration tanks from PETL (AER1 and AER2). For other abbreviations, please see materials and methods.

The bacterial diversity at phylum level in different WWTPs is represented in Fig 4. *Proteobacteria* was the dominant phyla in all the aeration tank samples. The samples from PETL had significantly lower bacterial diversity compared to other WWTPs (Student’s t-test, p = 0.0017) comparing the number of detected OTUs at similar sequencing depths (Table 3). Along with recycling of the sludge, around 20% w/v raw human feces is added to PETL for maintenance of bacterial diversity and activity [4, 5]. Still, the bacterial diversity in PETL is not near that of other sewage treatment plants. This suggests that incoming antibiotic containing waste strongly modulates the bacterial diversity.

**Table 3:** Diversity indices in samples from the aeration tanks of PETL (AER1 and 2) and from municipal sewage treatment plants (Zhang et al, 2012), based on 16S amplicon sequencing (16,770 sequences per sample).

Sample	Chao 1	Goods coverage	Shannon diversity	Simpson's diversity	Observed species	PD whole tree
CNGZDT	2954.9	0.952	9.15	0.995	2028	34.0
CNHKSH	3000.3	0.949	8.75	0.991	1980	35.5
CNBJBX	3429.1	0.944	9.33	0.995	2298	39.4
CNWHLW	2143.8	0.964	8.09	0.988	1457	26.1
CNHKSL	3915.4	0.932	8.82	0.969	2631	43.7
CNHKST1	4333.0	0.929	9.87	0.997	2773	43.4
CNSHMH	2253.2	0.963	8.03	0.987	1472	27.6
AER2	441.6	0.992	3.08	0.735	281	4.2
AER1	185.3	0.997	1.62	0.400	127	3.5

Abbreviations: AER1 = aeration tank No. 1; AER2 = aeration tank No. 2; CNGZDT, CNHKSH, CNBJBX, CNWHLW, CNHKSL, CNHKST1, CNSHMH = Aeration tanks from Chinese municipal treatment plants (details in S1 Table and Zhang et al. 2012)

The maintenance of a high bacterial functional diversity during AS treatment is important for functionality. Hernandez-Raquet et al, 2013 has shown that lower bacterial diversity of AS correlates to lower functional diversity in terms of the ability of the community to utilizing different carbon sources, as well as to the efficiency of AS treatment. The xenobiotic degradation and pollutant removal potential during AS treatment was limited when bacterial diversity was low [41]. In accordance, reports show that treated effluent from PETL consistently had total dissolved solids (TDS) and chemical oxygen demand (COD) higher than the prescribed emission limits [9] suggesting low removal efficiency of organic contaminants. Low bacterial diversity in PETL is likely to be one of the factors behind this. In environments exposed over longer times to chemical stressors, such as in the highly contaminated environments in Patancheru, it is possible that the microbial diversity could be partly restored, either because bacteria gain the ability to tolerate or even utilize the contaminants, or because sensitive species replace tolerant ones, or a combination of both processes [6, 38, 42].

## Conclusion

To the best of our knowledge, this is the first comprehensive study exploring the bacterial diversity in different sections of a full scale functional WWTP treating wastewater from multiple antibiotic production units. The study identifies dominating taxa and demonstrates clear changes in the bacterial community during different stages of the AS treatment process. The overall low bacterial diversity and high abundance of highly drug resistant bacteria suggests that antibiotic present in the waste modulates the microbial communities, which in turn might affect general treatment efficiency. Proper management of antibiotic production waste is hence necessary not only in order to reduce the risks for spread of antibiotics and antibiotic resistant bacteria, but also to reduce omissions of other organic contaminants.

## Supporting Information

S1 Fig Rarefaction curves for PETL samples. Abbreviations: EQR = equilibrator; AER1 = aeration tank No. 1; AER2 = aeration tank No. 2; STL = settling tank; SS = secondary sludge; DS = dewatered sludge; OS = old dried sludge
(TIF)

S1 Table Information regarding the WWTPs from a previous study published by Zhang et al, (2012). (DOCX)
