# Supplementary figures and images for: Limited Bacterial Diversity within a Treatment Plant Receiving Antibiotic-Containing Waste from Bulk Drug Production
Source: PLoS One. 2016 Nov 3;11(11):e0165914. doi: 10.1371/journal.pone.0165914 (PMC5094703; doi:10.1371/journal.pone.0165914)

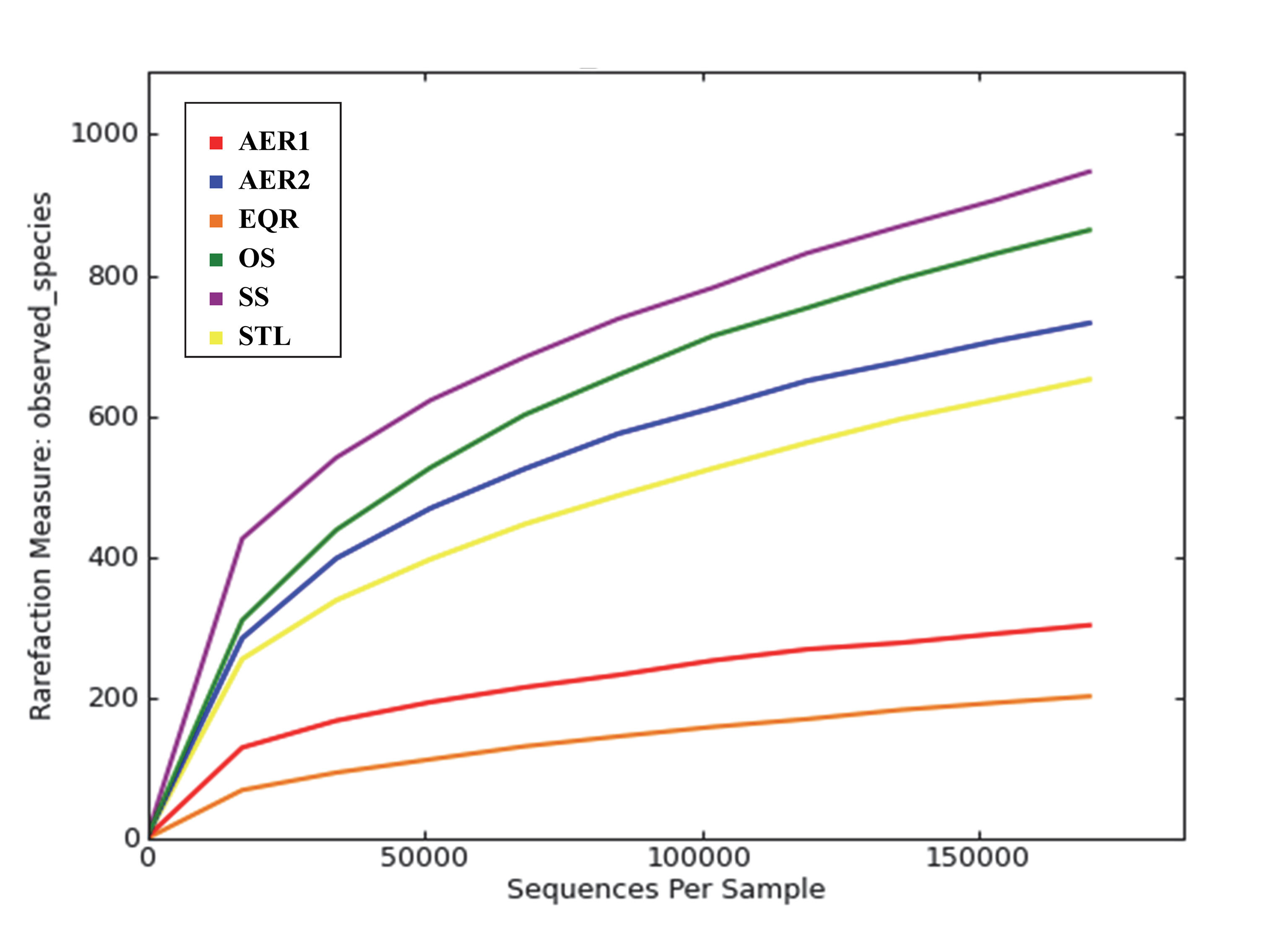

Supplement: S1 Fig — Abbreviations: EQR = equilibrator; AER1 = aeration tank No. 1; AER2 = aeration tank No. 2; STL = settling tank; SS = secondary sludge; DS = dewatered sludge; OS = old dried sludge (TIF) [file pone.0165914.s001.tif]
