# Supplementary material for: Limited Bacterial Diversity within a Treatment Plant Receiving Antibiotic-Containing Waste from Bulk Drug Production
Source: PLoS One. 2016 Nov 3;11(11):e0165914. doi: 10.1371/journal.pone.0165914 (PMC5094703; doi:10.1371/journal.pone.0165914)
Supplement: S1 Table — (DOCX) [file pone.0165914.s002.docx]

**Table S1**: Information regarding the WWTPs from previous study published by Zhang et al, (2012)

| Sample | Name of WWTP | City ,Country |
| --- | --- | --- |
| CNGZDT | Da-Tan-Sha | Guangzhou, PRC |
| CNHKSH | Shek-Wu-Hui | Hong Kong, PRC |
| CNBJBX | Bei-Xiao-He | Beijing, PRC |
| CNWHLW | Long-Wang-Zui | Wuhan, PRC |
| CNHKSL | Stanley | Hong Kong, PRC |
| CNHKST1 | Sha-Tin | Hong Kong, PRC |
| CNSHMH | Min-Hang | Shanghai, PRC |
